# The association between serum adropin and carotid atherosclerosis in patients with type 2 diabetes mellitus: a cross‑sectional study

**DOI:** 10.1186/s13098-022-00796-y

**Published:** 2022-02-08

**Authors:** Wen Wei, Hui Liu, Xiuping Qiu, Jushun Zhang, Jianqing Huang, Hangju Chen, Shuilin Qiu, Ruiyu Lin, Shihai Li, Mei Tu

**Affiliations:** 1 Department of Endocrinology, Fujian Longyan First Hospital, Longyan First Affiliated Hospital of Fujian Medical University, Longyan, 364000 China; 2 The Second School of Clinical Medicine, Southern Medical University, Guangzhou, 510515 China; 3 Department of Clinical Laboratory, Fujian Longyan First Hospital, Longyan First Affiliated Hospital of Fujian Medical University, Longyan, 364000 China; 4 Department of Endocrinology, Fujian Longyan First Hospital, Fujian Medical University, Fuzhou, 350004 China; 5 Department of Anesthesia, Fujian Longyan First Hospital, Longyan First Affiliated Hospital of Fujian Medical University, Longyan, 364000 China

**Keywords:** Adropin, Carotid atherosclerosis, Type 2 diabetes mellitus

## Abstract

**Background:**

Adropin, a newly‑identified energy homeostasis protein, has been implicated in the maintenance of metabolic homeostasis and insulin sensitivity. This study attempts to measure the association between serum adropin and carotid atherosclerosis in patients with type 2 diabetes mellitus (T2DM).

**Methods:**

This cross‑sectional study was performed in 503 hospitalized patients with T2DM.Serum adropin level was measured by a sandwich enzyme-linked immunosorbent assay. The carotid atherosclerosis was assessed by color Doppler sonography. The association between adropin and carotid atherosclerotic plaque was tested by logistic regression model. The effect of adropin on carotid intimal-medial thickness (CIMT) was estimated using linear regression model.

**Results:**

Overall, 280 (55.7%) patients had carotid atherosclerotic plaque. The risk of carotid atherosclerotic plaque decreased with the increment of serum adropin level (adjusted odds ratio [aOR], 0.90; 95%CI: 0.81–0.99) in patients with T2DM. Serum adropin (Standardized β = − 0.006, p = 0.028) was also independently protective factor for CIMT in patients with T2DM.

**Conclusion:**

In patients with T2DM, high serum adropin level was correlated with a decreased risk of carotid atherosclerosis in T2DM patients. Low circulating level of adropin may promote carotid atherosclerosis.

## Background

Cardiovascular disease (CVD), including coronary artery disease (CAD), stroke, and peripheral artery disease (PAD), is a well-known leading cause of mortality in diabetic patients [1]. Atherosclerosis is one of the major underlying factors [2]. Although metabolic disorder in diabetes has been proved to be an important mediator to initiate and promote atherosclerosis [3], there are still some potential related molecules that may affect the development of atherosclerosis in diabetes. Exploration of related molecules may provide new biomarkers or therapeutic targets for atherosclerosis in diabetes.

Adropin is a bioactive protein encoded by the energy homeostasis associated gene (Enho) that is expressed in the liver and brain. Adropin contains 76 amino acids and has a molecular weight of 4.5 kDa [4]. Adropin regulates various signaling pathways to enhance insulin sensitivity, glucose metabolism, endothelial function, and motor coordination. In neurons, adropin binds to contactin 6 to activate Notch1 signaling and regulate brain development. In addition, adropin activates mitogen-activated protein kinase (MAPK) signaling in endothelial cells through either vascular endothelial growth factor receptor 2 (VEGFR2) or in cardiomyocytes through G protein-coupled receptor 19 (GPR19) [5]. Adropin has an important role in maintaining energy homeostasis and insulin sensitivity, which has been proved to attenuated the development of atherogenesis in animal experiment [6–8]. Low serum adropin level was also associated with stable angina pectoris, acute myocardial infarction and the severity of coronary atherosclerosis [9–14]. Fujie S et al. also found a negative correlation between serum adropin level and carotid arterial stiffness [15]. However, no study has examined the relationship between circulating adropin level and carotid atherosclerosis in diabetic patients.

Therefore, we aim to assess the association between serum adropin and carotid atherosclerosis in patients with type 2 diabetes mellitus (T2DM).

## Methods

### Study population and data sources

Our study was cross-sectional and the data were extracted from the electronic clinical management records system of Longyan First Affiliated Hospital of Fujian Medical University. Totally, 503 adult patients (≥ 18 years of age) with T2DM hospitalized for either diabetic complications screening or poor blood glucose control were continuously observed from July 2018 to June 2019. Patients with ketoacidosis, hyperosmolar status, acute severe infection, renal diseases on hemodialysis, autoimmune disease, malignant cancer, severe cardiac insufficiency, and incomplete clinical parameters were eliminated. The study was approved by the ethics committee of Longyan First Affiliated Hospital of Fujian Medical University (approval number LY-2017–068), and written informed consent was obtained from all participants.

### Data collection

We recorded information about all participants, such as smoking habit, duration of diabetes, history of diabetic complications, hypertension, CAD and stroke, administered drugs including insulin or oral antidiabetic drugs (OADs), antihypertensive agents (AHAs), statins and aspirin, laboratory test results and other clinical variables including height, weight, waist circumference (WC) and blood pressure (BP). Venous blood samples were collected in the early morning after overnight fasting.

Hypertension was defined as current treatment for hypertension, or systolic blood pressure ≥ 140 mmHg or diastolic blood pressure ≥ 90 mmHg. Body mass index (BMI) was calculated by dividing weight (kg) by the square of height (m). Homeostatic model assessment-insulin resistance (HOMA-IR) was calculated using the following formula: fasting blood glucose (mmol/l) × fasting plasma insulin (mU/l)/22.5. Estimated glomerular filtration rate (eGFR) value was calculated based on serum creatinine (Scr) level using the Modification of Diet in Renal Disease (MDRD) formula.

### Measurement of adropin

Samples of venous blood were collected after overnight fasting, and stored at − 80˚C prior to analyses. Serum adropin level was measured by a commercial enzyme-linked immunosorbent assay (ELISA) kit (Nanjing Camilo biological engineering Co., Ltd., Jiangsu, China), which give intra-batch and inter-batch variations were 8% and 12%, respectively. The lowest level of adropin ELISA assay was 0.5 ng/ml. Duplicate measurements were obtained for all samples.

### Ultrasonography measurements

Ultrasonography was performed by two experienced sonographers under a standardized protocol. Color Doppler sonography was performed with a high frequency linear transducer (5–12 MHz, Epiq5, Philips Ultrasound, Bothell, WA). All subjects were examined in a supine position, with a slight rotation of the neck to the contralateral side with the minimal tension of the cervical muscles. Carotid arteries were examined bilaterally at the common carotid arteries, the bifurcation, the external carotid arteries, and the internal carotid arteries from transverse and longitudinal orientations and were scanned in the anterolateral, posterolateral and mediolateral directions to assess the presence of atherosclerotic plaque and stenosis and measure intima-media thickness (IMT).

Atherosclerotic plaque was defined as a focal thickening of the intima-media complex encroaching into the arterial lumen by at least 0.5 mm or involving 50% of the surrounding IMT, or a focal thickening from the intima-lumen interface to the media-adventitia interface of over 1.5 mm [16]. Carotid atherosclerotic plaque was defined as the presence of atherosclerotic plaques in any of the aforementioned carotid arteries segments [17, 18]. Carotid intimal-medial thickness (CIMT) is perceived as common carotid IMT, which is calculated as the mean of the single maximum CIMT measurements that are measured from different segments of the carotid artery. When plaques are present in a segment, the maximal value is by definition at the maximum height of the plaque [16]. Mean CIMT was defined as the mean values of bilateral CIMTs.

### Statistical analyses

For continuous variables, normality was checked. If the data showed a normal distribution, variables were given as mean ± standard deviation, and two-sample independent t-test was used to compare differences among groups. If the data were not distributed normally, the Mann–Whitney U non-parametric test was employed and variables were expressed as median with interquartile range. For categorical variables, they were expressed by absolute numbers and percentages. Chi-square test was used to evaluate the differences in categorical variables.

Restricted cubic splines were used to detect the association between the serum adropin level and carotid atherosclerotic plaque or mean CIMT. The relationship between adropin and carotid atherosclerotic plaque was assessed by univariable and multivariable logistic regression. The effect of serum adropin level on CIMT was estimated using linear regression model before and after adjusting for confounding factors. Variables decided to enter the multivariable model were carefully selected based on variables associated with known risk factors or variables with p-value < 0.05 in baseline or in univariable regression analysis.

All analyses were performed with R software (version 4.0.5; R Foundation for Statistical Computing, Vienna, Austria). A two-sided *p*-value < 0.05 indicated significance for all analyses.

## Results

### Clinical characteristics

Totally, 503 subjects including 297 men and 206 women were enrolled in this study. Overall, the prevalence rate of carotid atherosclerotic plaque was 55.7%. The subjects were divided into two groups based on with or without carotid atherosclerotic plaque. There were no significant differences in the serum adropin level between the two groups (20.5 ± 4.0 vs. 20.6 ± 3.7 ng/ml p = 0.763) (Table 1).

**Table 1:** Comparison of clinical characteristics between patients with and without carotid atherosclerotic plaque

Characteristic	Without carotid plaque	With carotid plaque	*p*-value
	(n = 223)	(n = 280)	
Demographic characteristics			
Age (years)	51.6 ± 9.8	61.5 ± 10.1	< 0.001
Male	133 (59.6)	164 (58.6)	0.880
WC (cm)	88.6 ± 9.2	89.9 ± 10.8	0.033
BMI (kg/m2)	23.9 ± 3.1	24.8 ± 3.6	0.004
Medical history and Clinical condition			
Smoking history	70 (31.4)	103 (36.8)	0.242
Hypertension	75 (33.6)	131 (46.8)	0.004
SBP (mmHg)	134.4 ± 18.5	139.3 ± 19.3	0.004
DBP (mmHg)	83.9 ± 12.2	80.9 ± 12.2	0.006
Duration of diabetes (years)	4 (0, 10)	8 (3, 11)	< 0.001
DR	23 (13.9)	59 (27.7)	0.005
DPN	72 (55.0)	103 (57.2)	0.752
DN	15 (9.3)	51 (24.4)	< 0.001
Stroke	5 (2.2)	17 (6.1)	0.062
NAFLD	116 (58.0)	117 (50.2)	0.128
Laboratory examination			
Adropin (ng/ml)	20.5 ± 4.0	20.6 ± 3.7	0.763
FBG (mmol/L)	9.2 ± 3.4	9.1 ± 3.5	0.852
2hPBG (mmol/L)	13.1 ± 4.8	13.2 ± 5.1	0.751
HbA1c (%)	10.1 ± 2.5	10.7 ± 2.7	0.056
HOMA-IR	13.8 (7.6, 27.7)	17.9 (8.7, 32.3)	0.023
TG (mmol/L)	2.2 ± 1.8	1.9 ± 1.5	0.057
TC (mmol/L)	5.0 ± 1.2	4.9 ± 1.2	0.192
HDL-C (mmol/L)	1.2 ± 0.4	1.3 ± 0.5	0.167
LDL-C (mmol/L)	3.2 ± 1.0	3.1 ± 1.1	0.305
eGFR (ml/min/1.73 m^2^)	124.0 ± 38.1	106.8 ± 36.0	< 0.001
UA (μmol/L)	344.8 ± 92.4	345.0 ± 91.3	0.981
hs-CRP (mg/L)	1.2 (0.6, 2.3)	1.4 (0.7, 2.9)	0.090
HCY (μmol/L)	10.6 ± 3.3	11.4 ± 3.8	0.018
Administered drugs			
Insulin	36 (16.1)	58 (20.7)	0.234
OADs	123 (55.4)	173 (62.5)	0.133
Statins	6 (2.7)	17 (6.1)	0.112

*WC* waist circumference, *BMI* body mass index, *SBP* systolic blood pressure, *DBP* diastolic blood pressure, *DR* diabetic retinopathy, *DPN* diabetic peripheral neuropathy, *DN* diabetic nephropathy, *CAD* coronary artery disease, *NAFLD* Nonalcoholic fatty liver disease, *FBG* Fasting blood glucose, *2 h PBG* 2 h postprandial blood glucose, *HbA1c* glycosylated hemoglobin, *HOMA-IR* homeostatic model assessment-insulin resistance, *TG* triglyceride, *TC* total cholesterol, *HDL-C* high density lipoprotein cholesterol, *LDL-C* low density lipoprotein cholesterol, *eGFR* estimated glomerular filtrationrate, *UA* uric acid, *hs-CRP* hypersensitive C-reactive protein, *HCY* homocysteine, *OADs *oral antidiabetic drugs

Compared with patients without carotid atherosclerotic plaque, patients with carotid atherosclerotic plaque were likely to be older (61.5 ± 10.1 years vs. 51.6 ± 9.8 years, *p* < 0.001). These patients demonstrated longer duration of diabetes [8 (3, 11) years vs. 4 (0, 10) years, *p* < 0.001] and higher incidences of diabetic nephropathy (DN) (24.4% vs. 9.3%, *p* < 0.001), diabetic retinopathy (DR) (27.7% vs. 13.9%, p = 0.005), and hypertension (46.8% vs.33.6%, *p* = 0.004) than patients without carotid atherosclerotic plaque. Patients with carotid atherosclerotic plaque showed higher BMI, WC, SBP, HOMA-IR and homocysteine (HCY) levels than patients without. The detailed patients’ clinical characteristics are listed in Table 1.

### Association between serum adropin and carotid atherosclerosis

**Fig. 1:**
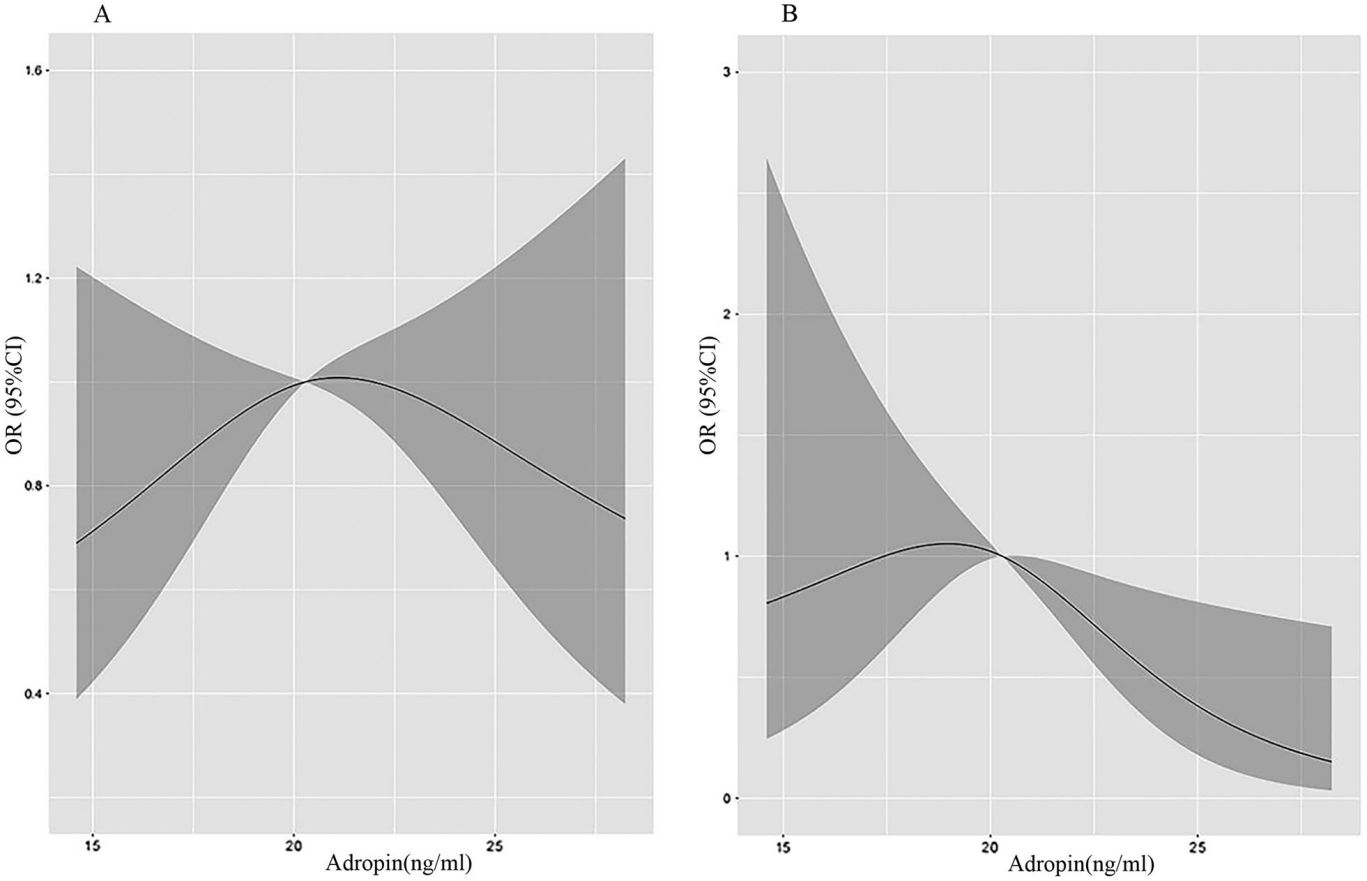
Restricted spline curve of the serum adropin level odds ratio of carotid atherosclerotic plaque. **A** The restricted spline curve of univariable logistic regression model. **B** The restricted spline curve of multivariable logistic regression model.

A linear association (Nonlinear *p* = 0.122) between carotid atherosclerotic plaque and serum adropin level was indicated in our study populations: the higher the serum adropin level, the lower the incidence of carotid atherosclerotic plaque (Fig. 1). The risk of carotid atherosclerotic plaque significantly decreased with the increment of serum adropin level (adjusted odds ratio [aOR], 0.90; 95% CI: 0.81–0.99) in patients with T2DM. Moreover, when the first tertile was used as the reference category, the adjusted association with risk was also significant for both the second and third tertile of adropin (Table 2).

**Table 2:** Logistic regression analysis for carotid atherosclerotic plaque

	Univariable		Multivariable	
	OR(95%CI)	*p*-value	aOR(95%CI)	*p*-value
adropin, continuous				
Per 1-point increment	1.01(0.96–1.06)	0.763	0.90(0.81–0.99)	0.034
adropin, categorical (Tertile 1 as reference)				
Tertile 2	1.15(0.75–1.78)	0.521	0.40(0.19–0.98)	0.039
Tertile 3	1.02(0.66–1.57)	0.936	0.30(0.12–0.74)	0.011

Adjusted for age, gender, smoke, BMI, WC, duration of diabetes, DN, hypertension, HOMA-IR, LDL-C, hs-CRP and HCY

*BMI* body mass index, *WC* waist circumference, *DN* diabetic nephropathy, *HOMA-IR* homeostatic model assessment-insulin resistance, *LDL-C* low density lipoprotein cholesterol, *hs-CRP* hypersensitive C-reactive protein, *HCY* homocysteine

**Fig. 2:**
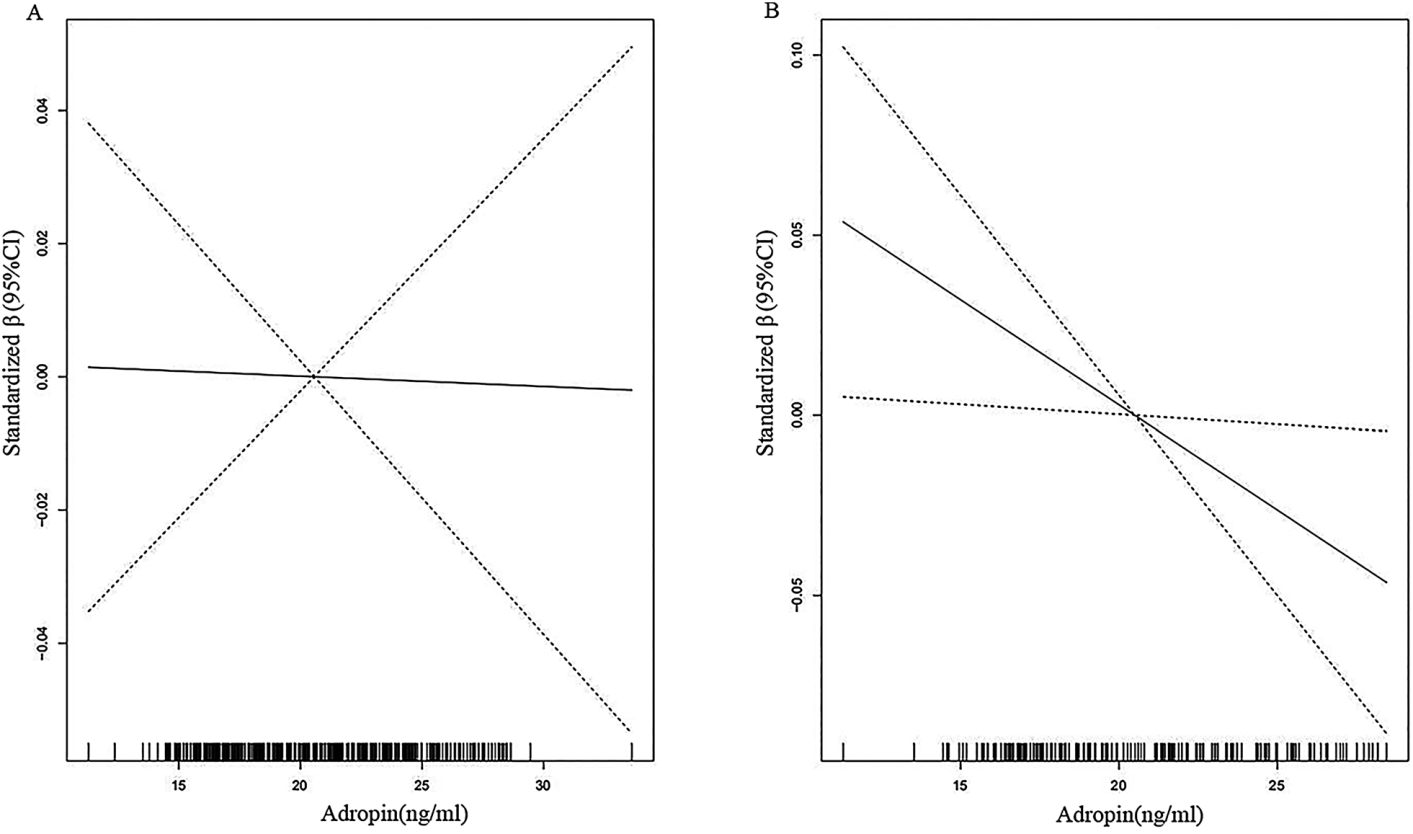
Restricted spline curve of the serum adropin level standardized β of mean carotid intimal-medial thickness. **A** The restricted spline curve of univariable linear regression model. **B** The restricted spline curve of multivariable linear regression model.

Restricted spline curve also showed that the higher the serum adropin level, the less the CIMT (Fig. 2). After adjusted for age, gender, smoke, BMI, WC, duration of diabetes, DN, hypertension, HOMA-IR, LDL-C, hs-CRP and HCY, linear regression analysis showed that serum adropin level (Standardized β = − 0.006, *p* = 0.028) was independently protective factor for CIMT in patients with T2DM (Table 3).

**Table 3:** Linear regression analysis for CIMT

	Univariable		Multivariable	
	Standardized β(95%CI)	*p*-value	Standardized β(95%CI)	*p*-value
Adropin, continuous				
Per 1-point increment	0.000(− 0.004 to 0.004)	0.938	− 0.006(− 0.011 to − 0.001)	0.028
adropin, categorical (Tertile 1 as reference)				
Tertile 2	0.005(− 0.032 to 0.041)	0.795	− 0.037(− 0.062 to − 0.002)	0.046
Tertile 3	− 0.015(− 0.052 to 0.022)	0.425	− 0.054(− 0.101 to − 0.008)	0.023

Adjusted for age, gender, smoke, BMI, WC, duration of diabetes, DN, hypertension, HOMA-IR, LDL-C, hs-CRP and HCY

*CIMT* carotid intimal-medial thickness, *BMI* body mass index, *WC* waist circumference, *DN* diabetic nephropathy, *HOMA-IR* homeostatic model assessment-insulin resistance, *LDL-C* low density lipoprotein cholesterol, *hs-CRP* hypersensitive C-reactive protein, *HCY* homocysteine

## Discussion

To our best knowledge, this present study was the first to evaluate the association between serum adropin and carotid atherosclerosis in patients with T2DM. The results showed that the risk of carotid atherosclerotic plaque and carotid artery intimal thickening decreased with the increment of serum adropin level. Low circulating level of adropin may be involved in promotion of carotid atherosclerosis.

It is well known that carotid atherosclerosis is a marker of systemic atherosclerosis and strong predictor of cardiovascular events [19–22]. Most seniors develop atherosclerosis as a function of age itself. Older diabetic patients with carotid atherosclerosis are often in high risk for CAD, PAD and/or cerebrovascular disease that further compromise functional capacity. The traditional risk factors such as age, smoke, diabetes, hypertension, dyslipidemia, obesity and insulin resistance are considered to be involved in the development of carotid atherosclerosis. Hcy and hs-CRP have been recognized as risk factors for atherosclerosis and cardiovascular diseases [23, 24]. Previous studies reported that low serum adropin level may be a risk factor or a potential predictor for developing coronary atherosclerosis [9–11]. Additionally, lower adropin level was associated with obesity, insulin resistance, hypertension and hs-CRP [7, 25–28]. Therefore, we adjusted for the known risk factors and statistically different factors, and then found the independently negative relationship between serum adropin level and the risk of carotid atherosclerosis. However, serum adropin levels did not differ significantly between groups and the univariable analysis was not statistically significant. These results may be partly explained by a relatively small population in our study. Another possible reason is that there is some correlation between adropin and confounding factors such as obesity, and the true effect of adropin is concealed by the effect of confounding factors. After eliminating the influence of confounding factors by multivariable analysis, the true effect of adropin on carotid atherosclerosis is revealed.

Atherosclerosis is a multifactorial and complex process involving endothelial dysfunction, vascular inflammation, vascular smooth muscle cells (VSMCs) proliferation, thrombus formation, monocytes infiltration and differentiation into macrophages, and the conversion of lesion-resident macrophages into foam cells [29]. The mechanism underlying the relationship between adropin and atherosclerosis may be as follows. Adropin is involved in the endothelial function and the inhibition of atherosclerosis by up-regulating endothelial nitric oxide synthase (eNOS) [30]. An in vitro laboratory experiment showed that endothelial cells treated with adropin exhibited greater proliferation, migration, capillary-like tube formation and up-regulation of the expression of eNOS [30]. Besides, the reduced circulating adropin concentration has an important correlate of metabolic disorders associated with insulin resistance and obesity, which are closely linked to the progression of atherosclerosis [6, 7, 31]. Adropin may be a potential anti-inflammatory protein and may play an important role in the prevention of atherosclerosis [32, 33].

Endothelial impairment and dysfunction caused by diabetic metabolic disorder has been confirmed as an important mediator in initiating and promoting atherosclerosis [3]. Our results further suggested that decreased adropin level will increase the risk of atherosclerosis in patients with T2DM. Since low serum adropin level and diabetes are both promoting atherosclerosis, their combination may lead to more severe atherosclerosis. Further studies are needed to investigate the causal relationship among adropin, diabetes and atherosclerosis.

Based on the mentioned studies it can be hypothesized that therapies addressing adropin could improve endothelial function, retard atherosclerosis, and decrease the risk for the development of insulin resistance. Fujie et al. also found aerobic exercise training increased the levels of serum adropin and plasma oxidase, and concomitantly reduced arterial stiffness [15]. Given that carotid atherosclerosis is associated with an increased risk of cardiovascular disease and low serum adropin level may be involved in promotion of carotid atherosclerosis, adropin may thus be a useful agent in preventing atherosclerosis and its progression. In addition, measurement of serum adropin level may allow clinicians to identify diabetic patients at elevated risk for carotid atherosclerosis.

Our study had several limitations. First, the cross-sectional design limited our ability to assess the cause-effect relationship between the serum adropin and carotid atherosclerosis. Second, this was merely a single-center study with a relatively small number of patients. The relationship between the risk of carotid atherosclerosis and adropin need to be confirmed in further larger prospective studies including nondiabetic population. Third, the precise regulatory mechanism associated with adropin and carotid atherosclerosis and whether adropin may be a useful agent in preventing atherosclerosis require further investigation.

## Conclusion

In the present study we found that the risk of carotid atherosclerosis decreased with the increment of serum adropin in patients with T2DM. Low circulating level of adropin may promote carotid atherosclerosis. Further studies revealing the immanent connection among adropin, diabetes and atherosclerosis may provide a novel biomarker for atherosclerosis in diabetic patients.

## Data Availability

Data relevant to this study are available from the corresponding authors upon reasonable request.

## Abbreviations

AHAs: Antihypertensive agents
ACEI/ARB: Angiotensin-converting enzyme inhibitor/angiotensin receptor blocker
BMI: Body mass index
CVD: Cardiovascular disease
CAD: Coronary artery disease
CIMT: Carotid intimal-medial thickness
CCB: Calcium channel blocker
DBP: Diastolic blood pressure
DR: Diabetic retinopathy
DPN: Diabetic peripheral neuropathy
DN: Diabetic nephropathy
ELISA: Enzyme-linked immunosorbent assay
eGFR: Estimated glomerular filtrationrate
Enho: Energy homeostasis associated gene
eNOS: Endothelial nitric oxide synthase
FBG: Fasting blood glucose
GPR19: G protein-coupled receptor 19
HbA1c: Glycosylated hemoglobin
HOMA-IR: Homeostatic model assessment-insulin resistance
HDL-C: High density lipoprotein cholesterol
HCY: Homocysteine
hs-CRP: Hypersensitive C-reactive protein
IMT: Intima-media thickness
LDL-C: Low density lipoprotein cholesterol
MAPK: Mitogen-activated protein kinase
MDRD: Modification of Diet in Renal Disease
NAFLD: Nonalcoholic fatty liver disease
OADs: Oral antidiabetic drugs
PAD: Peripheral artery disease
Scr: Serum creatinine
SBP: Systolic blood pressure
T2DM: Type 2 diabetes mellitus
TG: Triglyceride
TC: Total cholesterol
UA: Uric acid
VEGFR2: Vascular endothelial growth factor receptor 2
VSMCs: Vascular smooth muscle cells
WC: Waist circumference
2hPBG: 2 Hours postprandial blood glucose
